# Draft Genome Sequences of Two *Bacillus* spp. and an *Oceanobacillus* sp. Strain Isolated from Marine Macroalgae

**DOI:** 10.1128/MRA.01417-18

**Published:** 2019-02-14

**Authors:** Mugesh Subramanian, Murugan Maruthamuthu

**Affiliations:** a Department of Microbial Technology, School of Biological Sciences, Madurai Kamaraj University, Madurai, India; University of Arizona

## Abstract

We present the draft genome sequences of Bacillus flexus strain DMTMMB08, Bacillus licheniformis strain DMTMMB10, and Oceanobacillus picturae strain DMTMMB24, isolated from marine macroalgae.

## ANNOUNCEMENT

In marine environments, Bacillus sp. strains are common inhabitants and are known to produce a variety of natural products (1, 2). Bacillus flexus, Bacillus licheniformis, and Oceanobacillus picturae strains are known as alkali-halotolerant bacteria which produce bacteriocins, glycopeptides, exopolysaccharides, serine proteases, cellulases, and polyhydroxyalkanoates, and they are also known for their phosphate-solubilizing capabilities (3–11).

Bacillus flexus strain DMTMMB08 and Bacillus licheniformis strain DMTMMB10 were isolated from surface-sterilized tissues of Sargassum polycystum L., and Oceanobacillus picturae strain DMTMMB24 was isolated from surface-sterilized tissues of Acanthophora spicifera L. following the method of Stoltzfus et al. (12) and using Zobell marine agar (Himedia). After 72 h of incubation, individual bacterial colonies were transformed to liquid medium (Zobell marine broth) for overnight growth and then subjected to genomic DNA (gDNA) extraction and purification using a genomic DNA isolation kit (Qiagen). The sequencing library was set up with a TruSeq Nano DNA library preparation kit (Illumina) with gDNA, following the manufacturer’s instructions, and sequenced using a NextSeq 500 with 2 × 150-bp read chemistry (Eurofins Genomics, India). The sequenced raw data were processed to obtain high-quality clean reads using Trimmomatic version 0.38; reads with more than 10% quality threshold (QV) <20 Phred score and those <100 nucleotides (nt) in length were eliminated (13). After removing the adapters and low-quality sequences, 3,049,932 paired-end reads totaling 906,971,477 bases remained for Bacillus flexus strain DMTMMB08, 6,368,973 paired-end reads totaling 1,896,742,518 bases remained for Bacillus licheniformis strain DMTMMB10, and 6,532,483 paired-end reads totaling 1,945,935,542 bases remained for Oceanobacillus picturae strain DMTMMB24. *De novo* genome assembly was performed using the SPAdes genome assembler version 3.11.1 with default parameters (14). The resulting draft genome sequence consists of 189 contigs with a total genome size of 3,463,842 bp (38.1% GC content) for Bacillus flexus strain DMTMMB08, 24 contigs with a total genome size of 4,265,203 bp (45.9% GC content) for Bacillus licheniformis strain DMTMMB10, and 49 contigs with a total genome size of 3,663,112 bp (39.2% GC content) for Oceanobacillus picturae strain DMTMMB24, with average coverages of 257-fold, 416-fold, and 488-fold, respectively. The draft genome sequences were annotated using the NCBI Prokaryotic Genome Annotation Pipeline (15). A total of 3,638 (3,517 protein-coding genes), 4,469 (4,300 protein-coding genes), and 3,678 (3,563 protein-coding genes) genes were predicted in Bacillus flexus strain DMTMMB08, Bacillus licheniformis strain DMTMMB10, and Oceanobacillus picturae strain DMTMMB24, respectively.

Biosynthetic gene cluster (BGC) analysis was carried out using all analytical features of antiSMASH version 4.2.0 (16). Twenty-five BGCs from Bacillus flexus DMTMMB08, 33 BGCs from Bacillus licheniformis DMTMMB10, and 29 BGCs from Oceanobacillus picturae strain DMTMMB24 were predicted, respectively. All three bacteria were found to have the signature of known and putative BGCs, like type 3 polyketides (T3PKs), bacteriocins, siderophore synthases, terpenes, nonribosomal peptides (NRPs), polysaccharides, and fatty acids within them. In Bacillus flexus strain DMTMMB08, the novel hydroxamate siderophore signature was noted in the predicted BGCs. The presence of an ectoine BGC, with the capability of extreme osmotic tolerance, and a novel kijanimicin-like antibiotic BGC were found in Oceanobacillus picturae strain DMTMMB10. Further insights into these draft genome sequences will help guide natural product discovery from these strains in the future.

### Data availability.

This whole-genome shotgun project has been deposited at DDBJ/ENA/GenBank under the accession numbers QWLS00000000, QWLT00000000, and QWLU00000000 and the SRA accession numbers SRR8039988, SRR8039989, and SRR8039987 for Bacillus flexus DMTMMB08, Bacillus licheniformis DMTMMB10, and Oceanobacillus picturae strain DMTMMB24, respectively.
